# Closure of a Recurrent Urethrovaginal Fistula in a Girl with Cloacal Anomaly Using Deflux Injection

**DOI:** 10.1055/s-0038-1660805

**Published:** 2018-07-18

**Authors:** Hanan Said, Salahuddin S. Syed, Ali Zeinelabdeen, Mohamed Negm Fayez

**Affiliations:** 1 Department of Paediatric Surgery, International Medical Center, Jeddah, Jeddah, Saudi Arabia; 2 Department of Paediatric Surgery, Children Hospital, Leeds, United Kingdom of Great Britain and Northern Ireland; 3 Department of Paediatric Surgery, King Fahd Armed Forces Hospital, Jeddah, Western, Saudi Arabia

**Keywords:** cloaca, urethrovaginal fistula, use of Deflux

## Abstract

In a girl born with cloaca, both hemivaginae and rectum were located above the bladder neck, and both ureters were connected to the hemivaginae. After diverting colostomy and cystovaginoscopy on the second day of life, the repair of cloaca was performed at 10 months of age by posterior sagittal anorecto vaginoplasty (PSARVP), including laparotomy and bilateral ureteric reimplantation. Eight months after the surgery, she developed a vesicovaginal fistula, which was repaired and closed by open surgery through the bladder. Three months after this procedure, a tiny urethrovaginal fistula was noticed, which was closed at the age of 2 years using hook diathermy to refresh the edges and was then closed by Deflux injection. The proper closure of the urethrovaginal fistula was confirmed by radiology and cystoscopy 3 months after the surgery. This report shows that injection of Deflux into a tiny urethrovaginal fistula following refreshing the edges may be a valid treatment option in selected cases.

## Introduction

Cloacal anomaly is one of the rarest forms of anorectal malformation found in newborns, which has an incidence of 1 in 20,000 live births. 1 2 3 Apart from being at the most severe end of anorectal malformation spectrum, it also offers a formidable challenge to the surgeons. It requires the surgeon to have in depth knowledge of newborn female pelvic anatomy as well as considerable amount of specialized surgical skills to effectively and successfully treat patients with cloacal anomalies. 4 In spite of best interdisciplinary management and adequate surgical correction, the incidence of short- and long-term complications still remains high. The surgical correction needs to be tailored specifically for the patient, as minor variations in the anomaly does exist. 1 The complications arising also needs to be treated in a way which is patient specific. 5 The most acceptable approach for one-stage total correction of cloacal malformation is the combined posterior sagittal and abdominoperineal approach. 6 Urethrovaginal fistula is one of the difficult complications of surgical correction of cloaca.

## Case Report

**Fig. 1:**
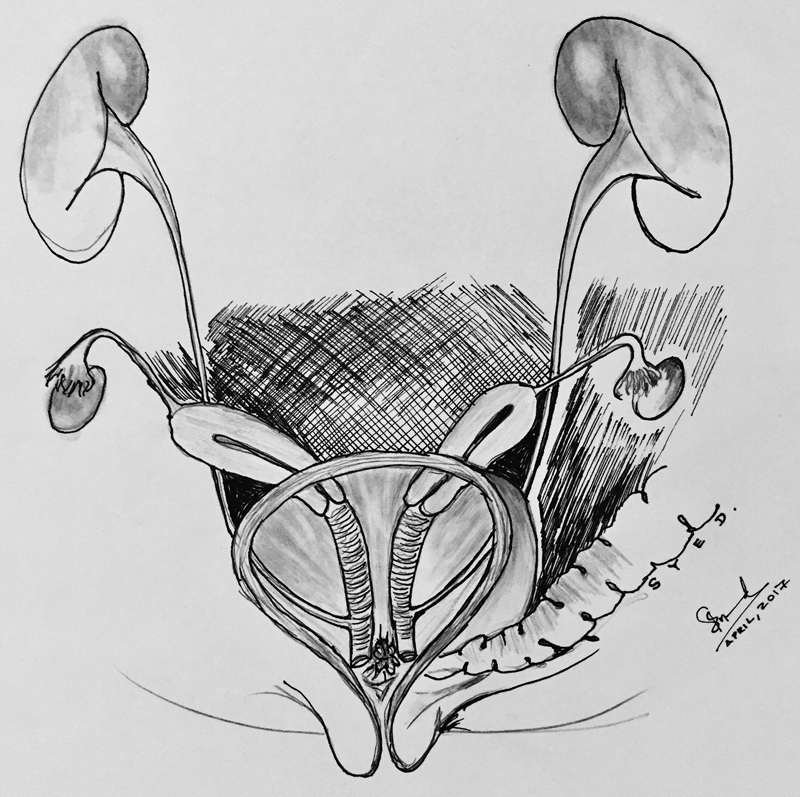
Common channel 3.5 cm at the second day of life.

**Fig. 2:**
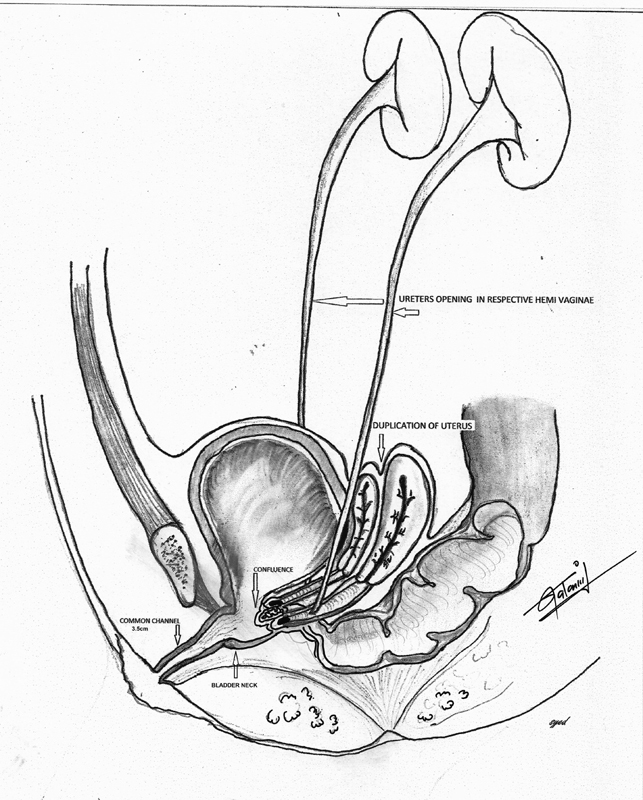
Duplicated uterus and two hemi-vagina with ectopic ureter.

**Fig. 3:**
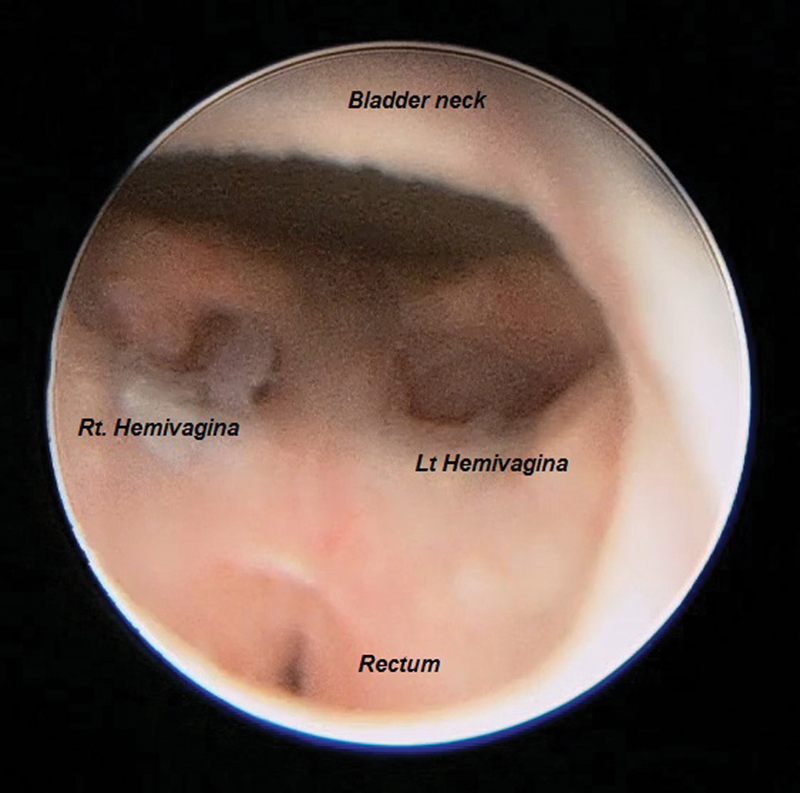
Cystoscopy view at the second day of life.

Our patient was born at term and was diagnosed at birth to have cloacal anomaly. She underwent diverting colostomy and cystovaginoscopy on the second day of her life where the common channel was 3.5 cm ( Fig. 1 ). It was found that she had duplication of uterus and vagina, and her both ureters were ectopically inserted into the corresponding hemivagina. The urinary bladder was empty and collapsed ( Fig. 2 ). The rectum and confluence of the hemivaginae opened above the bladder neck ( Fig. 3 ). The sacral ratio is 0.3. Full repair was done at the age of 10 months. Abdominal approach was utilized along with posterior sagittal anorecto vaginoplasty (PSARVP), where both ureters were reimplanted into the bladder, the common channel was left as urethra, the hemivaginae was combined and pulled down, and the rectum was pulled through the sphincter complex and repaired in the respective positions.

The post-operative cosmetic result was good, but the patient had urine leak through her vagina, which raised a suspicion of vesicovaginal fistula. This was confirmed by cystovaginoscopy where vesicovaginal fistula, ∼0.5 cm in diameter, was found. The fistula was approached through transvesical access and was closed in two layers without tissue interposition and catheter left for 10 days, 8 months after PSARVP. She eventually developed small urethrovaginal fistula, most likely as complication of vesicovaginal fistula repair. This tiny fistula, confirmed by cystoscopy, ∼4 to 4.5 cm distance from the skin, did not mandate a radical procedure for repair. According to our judgement, this would be marred with more harm than benefit to our patient.

**Fig. 4:**
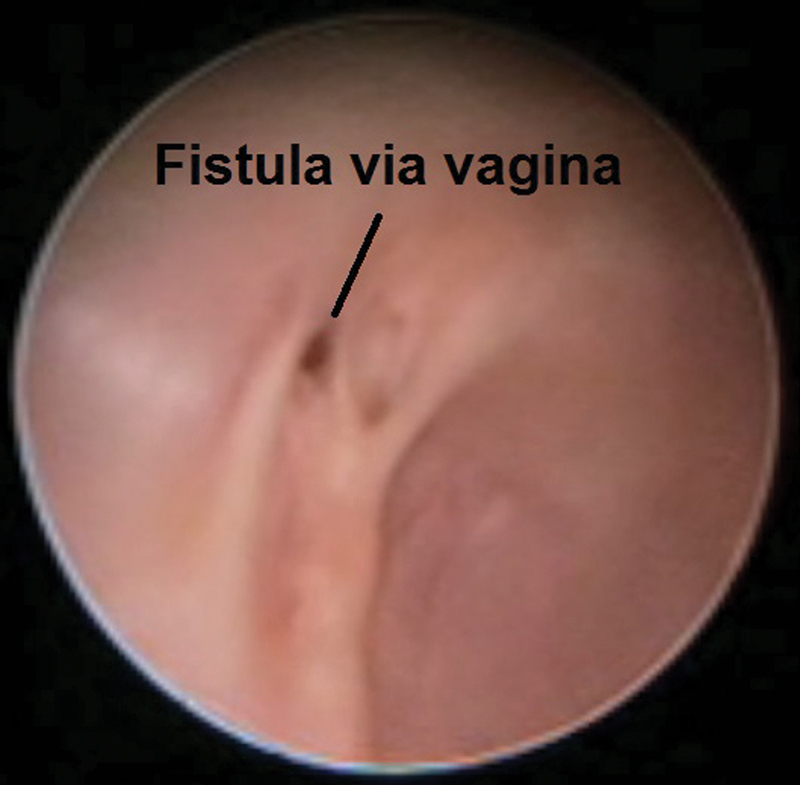
The uretherovaginal fistula via vagina.

**Fig. 5:**
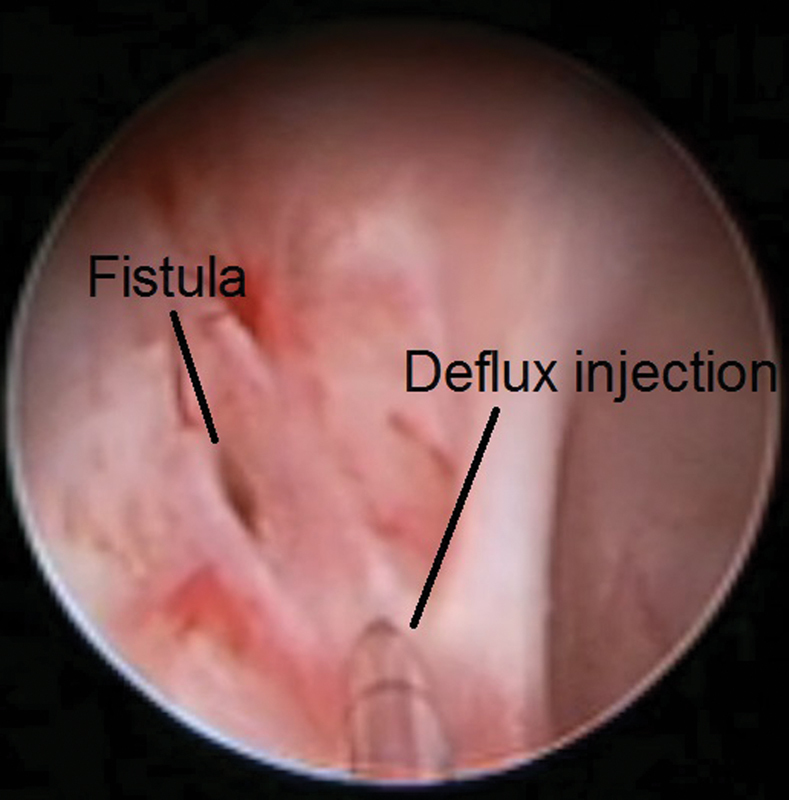
Deflux injection.

We devised a novel technique (full detailed explanation given to the family) where we approached the urethrovaginal fistula through a resectoscope via the vagina at 30 months of age. The edges were diathermised, and Deflux was injected just below the mucosa to approximate the diathermised edges of the fistula, and urinary catheter had been left for 5 days ( Figs 4 and 5 ). The new idea was discussed with an expert outside the hospital before the attempt.

The patient was referred postoperatively to the same center discussed before. The girl was passing urine spontaneously, and an ultrasound (US) showed normal kidney and no residual urine in the bladder.

Subsequent cystoscopy and vaginoscopy examinations did not reveal any recurrence of the urethrovaginal fistula. The patient was eventually examined by an experienced pediatric surgeon and pediatric urologist, and both ruled out any recurrence of the fistula.

Colostomy was closed at 3 years of age and started on CIC.

Because the mother was not compliant with CIC, MITROFANOFF was done for her at three and half years.

## Discussion

Management of complex urethrovaginal or vesicovaginal fistulae traditionally involves a radical approach where the fistulous tract is excised, and some vascularized graft or flap is interposed to prevent recurrence of the fistula. Even in the best hands, the rate of recurrence remains high due to the fact that urethra and vagina share a common wall, more so in cloacal anomalies, 7 and also due to compromised blood supply in an area that is breached by surgery. We had decided not to undertake a radical approach for the second time in this area, as further complication and redevelopment of fistula or complete breakdown of the repair was anticipated. Instead, a relatively simple and novel technique was adopted, which proved to be successful in closing the small fistula.

## Conclusion

The surgical and medical management of cloacal anomalies remains to have a high morbidity, such as the development of fistulas between the urethra and the vagina. If these are small, the injection of Deflux into the fistula may be a novel therapeutic option.

## Notes

New Insights and the Importance for the Pediatric Surgeon It is a unique technique to close a tiny fistula in difficult cases like urethrovaginal fistula post cloaca repair. It is a pioneer technique.
